# Role of Cilostazol Therapy in Hemodialysis Patients with Asymptomatic Peripheral Arterial Disease: A Retrospective Cohort Study

**DOI:** 10.1155/2016/8236903

**Published:** 2016-09-22

**Authors:** Paik Seong Lim, Yachung Jeng, Ming Ying Wu, Mei-Ann Pai, Tsai-Kun Wu, Chang Hsu Chen

**Affiliations:** ^1^Division of Renal Medicine, Tungs' Taichung MetroHarbor Hospital, Taichung, Taiwan; ^2^Department of Internal Medicine, Taipei Medical University, Taipei, Taiwan; ^3^Department of Rehabilitation, Jenteh Junior College of Medicine, Nursing and Management, Miaoli, Taiwan; ^4^Division of Biostatistics and Epidemiology, Department of Medical Research, Tungs' Taichung MetroHarbor Hospital, Taichung, Taiwan

## Abstract

*Background*. Peripheral arterial disease (PAD) and its relevant complications are more common in hemodialysis (HD) patients, while the evidence regarding antiplatelet therapy in CKD patients is scarce. We retrospectively analyzed the efficacy of cilostazol on outcomes in HD patients with asymptomatic PAD (aPAD).* Methods*. This cohort study enrolled 217 HD patients (median follow-up time: 5.75 years). Associations between cilostazol use and the outcomes were evaluated by time-dependent Cox regression analysis.* Results*. During follow-up, 39.5% (47/119) patients used cilostazol for aPAD and 31.8% (69/217) patients died. Cilostazol users had significantly lower CVD and all-cause mortalities (adjusted HR [95% CI]: 0.11 [0.03, 0.51] and 0.2 [0.08, 0.52]) than nonusers. Both death risks were nonsignificantly higher in cilostazol users than in HD patients without aPAD. The unadjusted and adjusted HR [95% CI] of CVD death risk were 0.4 [0.07, 2.12] and 0.14 [0.02, 0.8] for patients with aPAD during follow-up and were 0.74 [0.16, 3.36] and 0.19 [0.04, 0.93] for those with aPAD at initial.* Conclusions*. In HD patients with aPAD, lower CVD and all-cause mortality rates were observed in low-dose cilostazol user. Further evidences from large-scale prospective study and randomization trial are desired to confirm the effect of cilostazol.

## 1. Introduction

Peripheral arterial disease (PAD) a condition characterized by atherosclerotic occlusive disease of the lower extremities is commonly observed in patients with chronic kidney disease (CKD) patients, particularly those on dialysis [1–4]. The presence of PAD in CKD patients markedly increases the risk of amputation and cardiovascular disease (CVD) mortality, morbidity, and hospitalization compared to that of the general population [4, 5]. Indeed, the CVD mortality of patients with PAD is 4-5-fold higher than those without PAD [6]. Despite its importance, compared with CVD in other territories, there is less patient and physician awareness of its impact.

In general population, antiplatelet therapy is one of the main risk-reduction interventions recommended for PAD patients [7–9]. The benefit of a number of antiplatelet medications, including aspirin and clopidogrel, has been demonstrated in a wide set of studies of symptomatic individuals for whom a decreased risk of heart attack, stroke, and vascular death has been confirmed [9]. Nevertheless, clinical studies provide weak evidence of the efficacy of antiplatelet agents in preventing PAD progression [10, 11]. On the other hand, there is little information regarding the impact of antiplatelet agents on PAD outcomes such as amputation or cardiovascular outcome in patients with CKD. Until now, there are no randomized, controlled trials for the treatment of PAD in dialysis patients that establish the efficacy of any pharmacological agents. Hence, in this subgroup of patients, current intervention strategies are primarily based on the extrapolation of studies on the nonuremic counterparts. However, observational (CREDO) trial suggested that patients with CKD may not derive the same degree of benefit from clopidogrel therapy as those with normal renal function [12]. Furthermore, safety with antiplatelet therapy is a major concern, especially in CKD patients because of the potential for increased risk of bleeding events that might offset the potential benefit of reducing ischemic events. On the other hand, the utility of aspirin in lowering CVD events in patients with asymptomatic PAD remains unclear [13, 14]. Nevertheless, as per the K/DOQI clinical practice guidelines based upon these weak data, antiplatelet therapy is recommended for CKD patients with PAD to reduce the risk of overall cardiovascular events and death, unless contraindications exist.

Cilostazol, a quinolinone-derivative, selective phosphodiesterase (PDE) inhibitor, is a platelet-aggregation inhibitor and arterial vasodilator. This antiplatelet agent is used mainly for intermittent claudication in patients with peripheral artery disease [15]. Moreover, several controlled trials [16, 17] suggested that, compared with aspirin, cilostazol is associated with significantly less hemorrhagic stroke, the combined endpoint of stroke, myocardial infarction, and vascular death and total hemorrhagic events, with numerically less gastrointestinal bleed when used for the secondary prevention of stroke. In the absence of evidence to the contrary and apparently less bleeding risk, it might be reasonable to extend this therapy to the dialysis population. Currently, it remains unclear if cilostazol confers any clinical benefits in this vulnerable population. Here, we retrospectively analyzed the efficacy of cilostazol on outcomes in hemodialysis patients who suffered from asymptomatic PAD.

## 2. Patients and Methods

### 2.1. Study Population and Data Source

This was a retrospective, single-center study which was conducted in the Dialysis Center of Tungs' Taichung MetroHarbor Hospital (TTMHH) in the coastal region of central Taiwan. A cohort of 279 patients aged 20 or over, who have been on HD for at least 3 months prior to enrollment (January 1, 2008) was included. The medical charts of these patients were retrospectively reviewed for eligibility identification, of which 217 (78%) were compatible with the inclusion/exclusion criteria and enrolled in our analysis.

Medical charts were reviewed for information on mortality from enrollment through the end of the observation period (September 30, 2013). In this study, the diagnostic criterion for asymptomatic PAD was an ABI value lower than or equal to 0.9 with no clinical symptoms in the lower limb such as muscle discomfort or intermittent claudication. PAD was considered symptomatic if patients had ABI ≤0.9 and clinical symptoms or if they underwent previous surgical revascularization procedures or limb amputation. The characteristics of patients excluded from our study were (1) baseline ABI values > 1.3 (*n* = 2), (2) symptomatic PAD (*n* = 4), (3) decompensated cirrhosis (*n* = 3), (4) neoplastic diseases (*n* = 5), (5) incomplete data (*n* = 6), (6) receiving hemodialysis < 3 months (*n* = 7), (7) being transferred out before July 2008 (*n* = 17), (8) currently receiving antiplatelet therapy (*n* = 18), and undergoing a prior lower extremity vascular surgical revascularization procedure or transmetatarsal (below-the-knee or above-the-knee) amputation (*n* = 4). The baseline data such as demographics, comorbidities, anthropometrics, and relevant laboratory data, clinical diagnosis of PAD based on measurements of ABI, and medication history with antiplatelet drugs cilostazol of the 217 eligible cases were also retrieved. All patients were followed until death, loss to follow-up, end of observation (September 30, 2013), kidney transplantation, or transference to peritoneal dialysis or to other hospitals, whichever came first. The average period of postindex visit follow-up was 4.99 years (range 1.4–5.75 years). Patients alive by the end of follow-up or who died from non-CVD were censored cases in the survival analysis for CVD mortality. Those not experiencing death were censored cases in the survival analysis for all-cause mortality. CVD mortality included death caused by coronary artery disease, cerebrovascular disease, heart failure, and sudden death. Cilostazol was indicated for the prevention of ischemic vascular events in HD patients with PAD. Only those patients taking cilostazol medications for more than one year were identified as cilostazol users. Twenty-one patients were new users as they started cilostazol only during enrollment and 15 were taking cilostazol before enrollment. Their prescribed dosage was 50 mg twice a day. On the other hand, those HD patients with asymptomatic PAD whose medication use could not be retrieved from their medical records or those taking cilostazol medications for less than one year were considered as cilostazol nonusers.

### 2.2. Ankle Brachial Index Measurements

The ABI was measured by trained technicians using the Fukuda Vascular Screening System (VaSera VS-1000™, Fukuda Denshi Co., Ltd., Tokyo, Japan), which measures blood pressure from bilateral arm and ankle (brachial and posterior tibial arteries, resp.) simultaneously by an oscillometric method. The systolic pressure of the arm without dialysis access and the lower value of the ankle systolic pressure were used for the calculation. ABI was calculated by the ratio of the ankle systolic pressure divided by the arm systolic pressure. Of the two ABI values, respectively, calculated from the left- and right-limb measurements, the lowest value is used in this study. All participants were annually measured in a supine position after resting for at least 15 minutes and before dialysis.

In this study, ABI less than 0.90 was considered as evidence of PAD [18–20]. Absence of PAD was defined as ABI between 0.90 and 1.30 [21, 22]. Individuals with ABI greater than 1.30 were excluded, because this indicates poorly compressible leg arteries and inability to gauge arterial perfusion accurately [21, 23]. Of the 217 study cases, those with an initial ABI value ≤ 0.9 were identified as prevalent asymptomatic cases of PAD. For the rest, during the annual follow-up, those with any subsequent ABI values ≤ 0.9 were classified as incident asymptomatic cases of PAD cases. Patients who had serial ABI measurements above 0.9 during the entire observation period were considered as non-PAD group.

### 2.3. Ethics Statement

This study complies with the Declaration of Helsinki as well as its amendments and was performed after approval of the Institutional Review Board of TTMHH (number 103020). The written informed consent was waived after confirmation of the board since all study observations were retrospectively collected from regular health management records for maintenance HD patients, no invasive manipulations were involved in this study, and the data were analyzed anonymously.

### 2.4. Statistical Analysis

The descriptive statistics were expressed as mean ± standard deviation (SD), median with interquartile range (IQR), or frequency with percentage (%). The Kaplan-Meier method and log-rank test were applied to assess the survival functions. The time-dependent cox regression analysis was applied to assess the effect of cilostazol use on HD patients' survival, since patients of non-PADs initially could subsequently develop asymptomatic PAD and receive treatment during the follow-up period. The strength of the association between cilostazol use and outcomes was expressed as a hazard ratio (HR) with a 95% confidence interval (95% CI). The primary endpoints in this survival analysis were to assess if cilostazol use could confer any clinical benefits after adjusting other associated factors. Throughout this article, a significance level of 0.05 was applied in hypothesis tests for statistical association. A 95% confidence interval (CI) was listed whenever a hazard ratio (HR) was reported. All the statistical analyses were performed in SAS, version 9.1 (SAS Institute, Cary, NC, USA).

## 3. Results

### 3.1. Sample Characteristics

**Figure 1:**
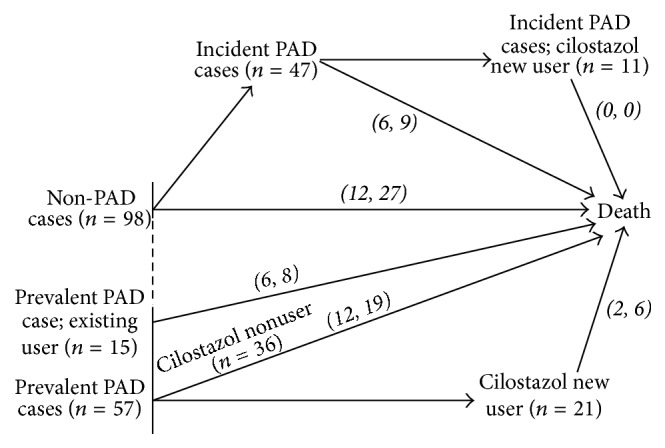
The diagram for the study sample distribution of the time-dependent covariates by the end of follow-up (*n* = 217). The* italic figures in parentheses* showed the death numbers of CVD and all-cause, respectively.

The summary of the study data was listed in Table 1. A total of 217 patients met the criteria for inclusion in this study; 197 (90.78%) had complete annual ABI measurements during their follow-up. Mean age was 62.9 ± 11.8 years; 49.32% were men (see Table 1). The prevalence of asymptomatic PAD initially was 33.18% (72/217); of the rest of the patients, 32.41% (47/145) patients were subsequently identified as asymptomatic PAD (incident cases) cases. From the medical records, 39.5% (47/119) patients used cilostazol under an indication of asymptomatic PAD. During the follow-up period, 38 (17.51%) patients died of CVD and the total number of deaths was 69 (31.8%).  Figure 1 showed the distribution of HD patient according to the PAD status and cilostazol usage by the end of follow-up.

### 3.2. Survival Analysis

**Figure 2:**
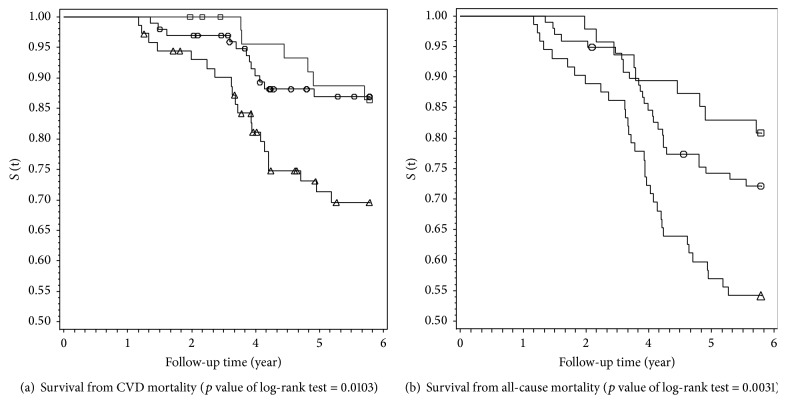
The Kaplan-Meier estimates for the survival functions of HD patients according to outcomes of PAD status during observation period. The triangle (△), square (□), and circle (○) on curves indicated censoring cases for prevalent PAD cases initially, incident PAD cases during follow-up, and non-PADs throughout follow-up.

**Figure 3:**
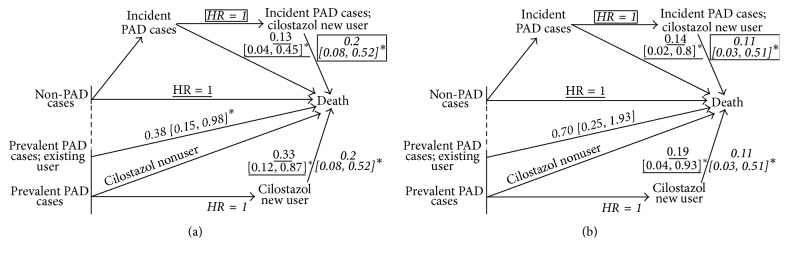
A diagram of the HRs for asymptomatic PAD and cilostazol use on all-cause (a) and CVD (b) mortality of HD patients (*n* = 217). The figures indicated beside arrows were HR [95% CI]. Asterisk (*∗*) indicates a statistically significant association. Labels “HR = 1” indicated the reference groups. Results of the same formats (italic with underlines, block letters, in parentheses, or italic with a frame) had identical reference group.

The Kaplan-Meier curves for the survival from CVD and from all-cause death according to the outcomes of PAD status by the end of observation period were displayed in Figure 2. Log-rank tests revealed the survival rates of prevalent PAD cases, incident PAD cases, and non-PADs were significantly different. The time-dependent cox regression analysis results for the asymptomatic PAD and cilostazol use were displayed in Figure 3 and Table 2.

The unadjusted and adjusted HRs for both CVD and all-cause mortality (Figures 3(a) and 3(b)) of cilostazol users against nonusers showed that the death risks significantly decreased after cilostazol use. The HRs for CVD death were 0.21 [0.05, 0.9] and 0.11 [0.03, 0.51] and those for all-cause death were 0.40 [0.16, 0.96] and 0.20 [0.08, 0.52]. More interestingly, the death risks of cilostazol users were not significantly higher than of non-PADs (unadjusted and adjusted HR ranged from 0.14 to 0.74 for CVD death risk and ranged from 0.13 to 1.01 for all-cause death risk). The *p* values for the above-mentioned HRs were listed in Table 2. In addition we also found that the differences of cilostazol use effect on death risks between incident and prevalent PAD cases were nonsignificant.

## 4. Discussion

In this study, we demonstrated the long term usage of the antiplatelet agents, cilostazol, in HD patients with asymptomatic PAD is associated with significant lower CVD and all-cause mortality. More interestingly, the death risk of HD patients with PAD who took cilostazol was not significantly higher than that of HD patients without PAD.

In the last few years, promising results from a number of studies showed that aspirin and cilostazol prevent recurrence of cerebral infarction [16, 17, 24, 25]. Several more recent meta-analyses showed that, compared with aspirin, cilostazol not only had similar or even slight better therapeutic effects in stroke prevention, but also had a significant reduction in the hemorrhagic stroke [18–21, 26]. It has been reported that Asians have a higher risk of recurrent ischemic and hemorrhagic stroke in the secondary stroke prevention phase [22]. Interestingly, a few studies and one recent meta-analysis revealed that cilostazol also provided a protective effect in the secondary prevention of the chronic phase of ischemic stroke in Asian patients [16, 20, 25]. The favorable effects of cilostazol in stroke prevention may be partly due to its pleiotropic effects or perhaps its ability to reduce artery stenosis. Apart from its antiplatelet effect, experimental studies suggested that cilostazol also exerts vasodilatory effect and increases human carotid, cerebral, coronary, and dermal blood flow [23, 27]. Besides, cilostazol was found to restore endothelial dysfunction and increase levels of vascular endothelial growth factor, which serves to repair damaged vascular epithelium [27, 28]. In several meta-analyses, the addition of cilostazol to antiplatelet therapy after peripheral vascular interventions is associated with improved outcomes [29–31]. In one report, the authors found that cilostazol may reduce long term (≥6-month) all-cause mortality by 31% over control in patients undergoing percutaneous coronary intervention [31]. In our cohort, despite the general comorbidity in the HD patients with asymptomatic PAD, cilostazol use may still confer survival benefit in these patients. Although the numbers of studied patients were small, we did observe that cilostazol provided similar efficacy in prevalent patients with PAD upon enrollment and incident PAD patients during follow-up. In contrast, for the subgroup of existing user, cilostazol appears to have less benefit. Although the reasons are not fully understood, timely and early introduction of cilostazol to asymptomatic patients seems reasonable.

Advanced CKD affects platelet function and coagulation cascade resulting in hemorrhagic tendencies and prothrombotic state [32, 33]. There is a clear dearth of studies evaluating the safety and efficacy of antiplatelet drugs for treating CKD patients. Hence, in CKD patients, the risks and benefits of antiplatelet drugs remain poorly defined. The researchers in the Dialysis Outcomes and Practice Patterns Study (DOPPS) showed aspirin prescription does not reduce the risk of cardiac events in patients on hemodialysis [34]. In addition, there have been no specific recommendations for antiplatelet therapy in HD patients with ischemic stroke.

The safety as well as efficacy balance of antiplatelet therapy in HD patients represents a crucial issue that may affect patient outcomes and contribute to underutilization of this group of medications. In a systemic review of 16 studies, the investigators found that out of total of 40,676 patients with ESRD only around 22% of patients are exposed to antiplatelet agent(s) [35]. Though two studies have addressed the safety of aspirin in secondary prevention of future cardiovascular events in CKD patients [35, 36], a more recent meta-analysis concluded that benefits for antiplatelet therapy among persons with CKD are uncertain and are potentially outweighed by bleeding hazards [37].

Together with aspirin and clopidogrel, cilostazol has been recommended as suitable options for the secondary prevention drugs of ischemic stroke by the American Heart Association and the American Stroke Association. However, the outcomes of cilostazol in CKD patients are not currently available. The starting or maintenance dose of cilostazol is often lowered in individuals with decreased kidney function (eGFR < 25 mL/min/1.73 m^2^), primarily to lower adverse-event rates such as headache, palpitation, or diarrhea. In this retrospective cohort, with lower dosage we found that cilostazol is safe and well tolerated. Interestingly, despite lower dosage, cilostazol significantly reduced both the CVD and all-cause mortality in those patients with cilostazol use when compared to those without. Moreover, in this analysis we did not find any major bleeding event in patients receiving cilostazol requiring hospitalization from our medical record retrieval. Overall, we feel that selection of an antiplatelet therapy should be individualized on the basis of patient risk factor profiles, tolerance, and other clinical characteristics. Clearly further studies and analyses are needed to clarify the exact role of this agent in this group of patients with high risk of future cardiovascular death especially cerebrovascular disease.

Previous studies assessed association between ABI level and mortality of CKD patients with only Kaplan-Meier curves comparison or only baseline ABI measurement considered in statistical models [5–9]. Many would agree that multiple measurements could reduce the chance of false diagnosis, as a sole technique was used for disease screening. The follow-up periods of most of the previous studies were of limited duration. Only very recently a Japanese long term cohort demonstrated that a higher linear-fitted decline rate of ABI and a lower baseline ABI were risk factors for CVD morbidity and mortality among patients on hemodialysis [38]. With annual measurements of ABI, our study clearly demonstrated the significance of persistent low ABI levels (<0.9) for CVD and all-cause mortalities without being based on a linearity assumption.

This study also has several potential limitations that remain to be addressed. First, this is a retrospective observational analysis with a small sample size. The limitations inherent to retrospective follow-up study are applicable to this study. Second, single-institution series are often biased towards particular patient demographics and practice patterns, but these data represent the real-world application of cilostazol in high-risk HD patients with PAD. Lastly, patients with ABI below 0.9 were considered positive for PAD for non-CKD patients but the accuracy of ABI assessment in CKD populations needs further verification. Using duplex ultrasound, Ogata et al. [39] found that ABI measurement might be less sensitive for detecting PAD in diabetic, elderly, or malnourished HD patient. However, in our cohort, duplex ultrasound was not routinely performed in these asymptomatic patients; thus the detailed information of lumen loss was unavailable and may underestimate the presence of PAD in HD patients.

In conclusion, our retrospective study demonstrated that HD patients with asymptomatic PAD had lower CVD and all-cause mortality rates after low-dose cilostazol was administered. Clearly, regular surveillance with ABI will allow earlier identification of cases at greater risk of developing PAD. More importantly, early administration of cilostazol may confer survival advantages to these patients. Clearly, more convincing data on the safety and efficacy of the agent for treating HD are still lacking and further trials are warranted to objectively evaluate their efficacy in this vulnerable group of patients.

## Figures and Tables

**Table 1:** Clinical characteristics of patients (*n* = 217).

Variables^†^	*n*	%	Mean ± SD	Median (IQR)
*Baseline profiles*				
Age (years)			62.88 ± 11.75	62 (55, 72)
Sex (male)	108	49.32		
HD vintage (years)^(5)^			4.2 ± 4.5	2.84 (0.74, 5.98)
BMI (Kg/m^2^)			23.14 ± 3.67	22.68 (20.7, 25)
SBP (mmHg)			137.7 ± 23.03	138 (120, 155)
DBP (mmHg)			76.53 ± 12.15	77 (68, 86)
*Past history *				
DM	103	47.03		
HTN	152	69.41		
CAD	89	40.64		
CeVD	31	14.16		
CHF^(3)^	22	10.05		

*Biochemical measurements*				
Alb (g/dL)			4.08 ± 0.32	4.1 (3.9, 4.3)
Ca^(1)^			9.54 ± 0.63	9.6 (9.1, 10)
Chol (mg/dL)^(1)^			171.16 ± 39.31	167 (142, 195.5)
CO_2_ (mEq/L)^(1)^			25.84 ± 2.74	25.95 (23.95, 27.85)
FBS (mg/dL)			112.42 ± 44.58	94 (84, 129)
Ferritin (*μ*g/dL)			558.18 ± 281.25	514 (381, 678)
Hb (g/dL)			11.26 ± 1.62	11.1 (10.2, 12)
hsCRP (mg/L)			4.72 ± 4.37	3.5 (1.7, 6.3)
HDL-C (mg/dL)^(1)^			46.08 ± 15.48	43.5 (35, 56.5)
LDL-C (mg/dL)^(2)^			93.9 ± 32.78	89.6 (72.8, 118)
TG (mg/dL)			157.83 ± 113.4	130 (86, 193)

Note: ABI: ankle brachial pressure index. Alb: albumin. BMI: body mass index. Ca: serum calcium. CAD: coronary artery disease. CeVD: cerebrovascular disease. CHF: congestive heart failure. Chol: total cholesterol. CO_2_: blood carbon dioxide. DBP: diastolic blood pressure. DM: diabetes mellitus. FBS: fasting blood sugar. Hb: hemoglobin. Hct: hematocrit. HD: hemodialysis. HDL-C: high density lipoprotein cholesterol. hsCRP: high-sensitivity C-reactive protein. HTN: hypertension. IQR: interquartile range. LDL-C: low-density lipoprotein cholesterol. P: serum phosphorus. PLT: platelet count. SBP: systolic blood pressure. SD: standard deviation. TG: triglyceride.

†: The superscripts (1), (2), (3) and (5) in this column indicate the numbers of missing values of the variables.

**Table 2:** The multiple cox regression analysis results for mortalities of HD patients (*n* = 217).

Death causes	CVD				All-cause			
Variable	HR	95% CI		*p* value	HR	95% CI		*p* value
*Time-dependent covariates*								
Incident PAD cases with cilostazol user (new)								
Versus cilostazol nonuser	0.11	0.03	0.51	0.0045^**∗**^	0.21	0.08	0.52	0.0008^**∗**^
Versus non-PAD group	0.14	0.02	0.8	0.0271^**∗**^	0.13	0.04	0.45	0.0013^**∗**^
Prevalence of PAD cases with cilostazol user (new)								
Versus cilostazol nonuser	0.11	0.03	0.51	0.0045^**∗**^	0.21	0.08	0.52	0.0008^**∗**^
Versus non-PAD group	0.19	0.04	0.93	0.0397^**∗**^	0.33	0.12	0.87	0.0258^**∗**^
Prevalence of PAD cases with cilostazol user (existing)								
Versus cilostazol user (new)	6.16	1.23	30.96	0.0272^**∗**^	1.89	0.62	5.8	0.2665
Versus cilostazol nonuser	0.7	0.25	1.93	0.4851	0.38	0.15	0.98	0.0451^**∗**^
Versus non-PAD group	1.19	0.41	3.51	0.7475	0.61	0.23	1.64	0.3321

*Covariates at baseline*								
DM, yes versus no	2.67	1.19	6	0.0172^**∗**^	2.53	1.39	4.62	0.0024^**∗**^
CAD or CeVD present, yes versus no	8.78	3.43	22.49	<0.0001^**∗**^	4.7	2.49	8.87	<0.0001^**∗**^
DBP, ≤68 or >86 versus others (mmHg)	2.59	1.27	5.32	0.0092^**∗**^	3.19	1.82	5.59	<0.0001^**∗**^
SBP, ≤120 versus others (mmHg)	—	—	—	—	2.91	1.62	5.23	0.0003^**∗**^
BMI, >22.83 versus others (Kg/m^2^)	0.37	0.19	0.75	0.0056^**∗**^	0.32	0.19	0.55	<0.0001^**∗**^
Albumin, ≥3.5 versus others (g/dL)	0.09	0.02	0.32	0.0003^**∗**^	0.18	0.07	0.5	0.0009^**∗**^
HD vintage, 10~20 versus ≤10 (years)	—	—	—	—	0.14	0.03	0.61	0.009^**∗**^

Note: see the footnotes of Table 1 for abbreviations and definitions of longitudinal PAD status patterns. The dichotomous cut-off points for BMI were the median and the first quartile; those for DBP and hsCRP were the first and third quartiles.

The asterisks “*∗*” indicate that statistically significant associations between outcomes and explanatory variables were observed at a 0.05 significance level.
